# Supplementary material for: Performance evaluation of micro lens arrays: Improvement of light intensity and efficiency of white organic light emitting diodes
Source: PLoS One. 2022 May 27;17(5):e0269134. doi: 10.1371/journal.pone.0269134 (PMC9140251; doi:10.1371/journal.pone.0269134)
Supplement: Attachment — Submitted filename: Plos One_Response to Reviewers.docx [file pone.0269134.s001.docx]

**Response to the Reviewers on PONE-D-22-07734**

Apurba Adhikary, Joy Bhuiya, Saydul Akbar Murad, Md. Bipul Hossain, K. M. Aslam Uddin, MD Estihad Faysal, Abidur Rahaman, and Anupam Kumar Bairagi

May 08, 2022

Dear Editor and Reviewers,

We want to thank the editor and all reviewers for their constructive comments, which have significantly improved technical content and presentation quality of the manuscript. We very much appreciate the reviewers’ and editor’s time and efforts. In addition, we have taken into consideration all comments and suggestions in the revised manuscript. Additionally, we have added an affiliation of the first author (Apurba Adhikary) as he started his PhD program at Kyung Hee University, South Korea. In the response letter, we have also described how we have addressed all comments and the changes that we have made. Some of the issues that are related to the further improvement and clarification of the revised manuscript are as follows:

- We have added the overall complexity in the revisited manuscript as per the suggestion of Reviewers.
- We have clarified and modified other questions that are raised by Reviewers.

Note:

- The reviewers’ comments are presented in italic font followed by our corresponding response.
- For the reviewers’ convenience, all revised texts and changes in this response letter and the revised manuscript are highlighted in blue text.
- All reference numbers in this response letter follow the section References at the end of this response letter, unless stated otherwise.

Best Regards,

The authors

**REVIEWER #1**

**Reviewer #1 comment 1:**

*OLED light extraction technology using MLA is a rather outdated topic. Related theories are known.*

**Response:**

We would like to thank the reviewer for pointing out this issue. We agree with the reviewer about OLED light extraction technology and related theories. The application of neuronal device equipped with an OLED requires strong luminescence well over the neuronal excitation threshold in addition to flexibility to enable the cell type-specific simulation to neurons [1]. The external quantum efficiency (EQE) of fluorescent OLEDs is limited to 5% due to the inability to use the generated triplet excitons whereas [2] report a blue fluorescent OLED that realizes an EQE as high as 14.1% by using a thermally activated delayed fluorescent (TADF) material as an assistant host in the emitting layer, which permits the efficient energy transfer of all generated excitons to the blue fluorescent dopant. Introducing gratings onto the metallic electrode is especially crucial for recovering the power lost to the associated SPP modes and [3] demonstrated the efficient outcoupling of SPP modes in TE mode by two-dimensional (2D) grating, which cannot be excited in one-dimensional (1D) grating OLED, and therefore increases efficiency up to 62.5% from 2D grating OLED than 1D grating OLED. On the other hand, we focused on improving the light intensity and efficiency of white organic light emitting diodes (OLEDs) by engraving micro lens arrays (MLAs) on the outer face of the substrate layer. In addition, we incorporated spherical and elliptical (planoconvex and planoconcave) MLAs to increase the light intensity and external quantum efficiency (EQE) of the white OLEDs. Therefore, our method increased the light intensity and EQE of the OLED and produced white light. For example, Convex-1 MLA engraved OLED showed an improvement of 3.8 times on the peak electroluminescence (EL) light intensity compared to basic OLED. We also compared the performance analysis of different micro lenses for increasing the light intensity and efficiency of white OLEDs. Finally, we also tried to represent the related theories in a new style but maintained the originality of the theories of OLED. We hope that reviewer will be satisfied with our explanation.

**Reviewer #1 comment 2:**

*In addition this paper only deals with simulation without providing actual device data.*

**Response:**

We would like to thank the reviewer for the observation and comment. We know that actual device data (experimental results) will give more clear idea about the performance of the designed OLEDs than the simulation results. Unfortunately, we don’t have any infrastructure to fabricate OLEDs in our laboratory. We will try to fabricate such kind of OLEDs in future, depending on the availability of appropriate equipment. However, goal of simulations is to predict physical properties from chemical composition, also known as the forward problem; this would allow for pre-screening and an overall better insight. Simulations can not only be advantageous but also are an essential tool in the design of future OLEDs [4]. We hope that the reviewer will understand our limitation and consider the simulation works for publication in the journal.

**Reviewer #1 comment 3:**

*"Acknowledgments" part is nonsensical.*

**Response:**

We thank the reviewer for pointing out this issue. We are really sorry for this unintentional mistakes. It was kept untouched and was copied from template. We have removed this section from the revised manuscript.

**Reviewer #1 comment 4:**

*This submission does not contribute to the field of OLEDs.*

**Response:**

We would like to thank the respected reviewer for this contractive comment. In [5], authors introduced an ultrathin silver (Ag) film as transparent electrode and conducted systematic modal analysis of OLEDs and reported that waveguide mode can be completely eliminated by designing an OLED structure that is below the cutoff thickness of waveguide modes. They also experimentally verified the waveguide mode removal in organic waveguides with the help of index-matching fluid and prism.  A research group reported that a blue fluorescent OLED that realizes an EQE as high as 14.1% by using a thermally activated delayed fluorescent (TADF) material as an assistant host in the emitting layer, which permits the efficient energy transfer of all generated excitons to the blue fluorescent dopant [2]. However, we focused on increasing light intensity and efficiency of OLEDs by engraving MLAs on the outer face of the substrate layer. The addition of MLAs on the substrate layer improves the light intensity and external quantum efficiency (EQE) of the OLEDs. In addition, four types of lenses (spherical and elliptical i.e., planoconvex and planoconcave) were incorporated on the outer face of the substrate layer to increase the EQE of the OLEDs. The maximum EQE of 17.30% was obtained for Convex-1 (elliptical planoconvex) MLA engraved OLED where the ERI was 1.70. Therefore, Convex-1 MLA incorporated OLED can be considered as a potential white OLED because of its excellent light distribution and intensity profile. We hope that our explanation will satisfy the reviewer. In addition, we also add some information based on the comments of the reviewer.

[Added Text] [Section “Materials and Methods”, page 6, lines 203-208 (Revised manuscript)]

“As stability and efficiency of OLED device is important, we achieved the optimized stability and efficiency by considering α-NPD and Alq_3_ as the donor i.e., electron-contributing and acceptor i.e., electron-accepting material in the emissive layer of OLEDs, correspondingly. Thus, we believe that our designed OLED will be stable and provides maximum performance which will lead to a great contribution in the field of OLEDs.”

**REVIEWER #2**

**Reviewer #2 comment 1:**

*In this manuscript, the author proposes a unique method to improve light intensity and efficiency of white organic light emitting diodes (OLEDs) by engraving micro lens arrays (MLAs) on the outer face of the substrate layer. This work is meaningful. However, there are several points that need to be clarified before the paper can be accepted for publication.*

**Response:**

We would like to express our sincere gratitude to the reviewer for appreciating our work and the positive comments. In our revised manuscript, we have carefully investigated concerns that are raised by the reviewer and modified accordingly.

**Reviewer #2 comment 2:**

*This work mainly talks about the impact of MLA on the efficiency and light intensity of OLED devices, but rarely explains the reasons for the impact of MLA on OLEDs, especially in the introduction, although many examples are listed, the essential reasons for the impact of MLA on OLEDs should be highlighted. Why does MLA improve device efficiency? It would be better if we could clearly explain the essential impact of MLA. Please comment it.*

**Response:**

We would like to thank the respected reviewer for this constructive comment. We understand the reviewer’s comment that we need to provide further revision and clarification regarding this issue. This work represented the impact of MLA on the efficiency and light intensity of OLED devices and also explain the reasons for the impact of MLA on OLEDs i.e., essential reasons for the impact of MLA on OLEDs in the “Introduction” section (lines 31-41) and “Performance Evaluation of MLA engraved OLEDs based on Physical Parameters” section (section 3.4; lines 542-552, and lines 553-561). As suggested by the reviewer, we also add the essential reasons for the impact of MLA on OLEDs in the “Introduction” section (lines 93-106).

**Action by the authors:**

In our revised manuscript, we have carefully revisited this part and modified accordingly. We are writing the mentioned lines for the reasons of impact of MLA on OLEDs in our manuscript for the consideration of reviewer as follows:

[Old Text] [Section “Introduction”, page 2, lines 27-37]

“The conventional OLEDs experiences certain problems including lower light extraction or out-coupling efficiency and minor viewing angle [5]. The extreme volume of light is missing inside the OLED device owing to various mechanisms which are responsible for the trapping of light and lower light extraction efficiency of OLED as shown in Figure 1 [6]. In addition, about 30% and 20% of light can be trapped due to the surface plasmons meaning inorganic layers and anode/organic waveguide modes in the anode layer [6]. Another major trapping of light (about 30%) is detected at the air/substrate interface owing to the TIR (total internal reflection) which causes the confinement of generated lights inside the OLED organic layers and in the interface of the substrate and transparent conducting oxide. For all the reasons, a small fraction of light is emitted in the air from the OLED device.”

[Old Text] [Section 3.4, pages 21-23, lines 511-521, lines 522-530]

“Therefore, it is important to select the proper OLED structure for commercial applications. The addition of MLA on the substrate layer facilitates the emitted lights to interfere with one another and generate constructive patterns and destructive patterns in different locations. The constructive and destructive patterns and distance between them determine the light spreading and smoothness of the OLEDs. The shape and nature of the micro lens array have an impact on these patterns. Although, the smoothness of light can be increased with the constructive interference pattern which is located close to one another, but any direct relationship between physical parameters of MLA and light distribution is not feasible. As mentioned before, elliptical MLA (Convex-1) engraved OLEDs showed higher peak EL intensity and EQE compared to spherical MLA (Spherical-7) engraved OLEDs, which was caused because of lens aberration effect [7]. The generated light of the OLED reflected back into the OLED device from the air/substrate interface because of total internal reflection (TIR). This phenomenon occurred since air has lower refractive index compared to substrate. Therefore, micro lens array is engraved on the substrate layer to reduce the TIR. OLED light extraction efficiency can be improved and the critical angle for TIR can be changed using MLA. For the MLA etched OLEDs, most of the incident angles of light were smaller than the critical angle, making it possible to add curvature lenses to the substrate layer, which led to most of the generated light being refracted from the outside surface. Consequently, light extraction efficiency i.e. EQE of the MLA engraved OLEDs was increased.”

[Added Text] [Section “Introduction”, page 3, lines 93-106 (Revised manuscript)]

The produced OLED lights reflected backwards onto the OLED device from the interface of air/substrate because of TIR. This kind of behavior of OLED lights occurred since substrate has higher refractive index compared to air. Consequently, we engraved micro lens arrays on the substrate layer to reduce the TIR. The incorporation of MLAs on the outer surface accelerates the emitted lights from the surface to intrude with each other and create constructive patterns and destructive patterns in numerous locations. These patterns and distance between them reveal the smoothness and light spreading of the generated OLEDs. Therefore, various shapes and sizes of micro lenses have different impacts on the constructive and destructive patterns. In addition, for the amalgamation of MLAs on the OLEDs, maximum incident angles of light became smaller compared to the critical angle because of curvature shape of the lenses on the substrate layer and led the generated light to be refracted from the outer surface. As a result, EQE and intensity of OLED lights can be increased.

**Reviewer #2 comment 3:**

*At the end of line 31 in the introduction, it should be clear whether the captured light is 30% or 20%, or between 20% and 30%.*

**Response:**

We would like to thank the reviewer for pointing out this unclear sentence. In the revised manuscript, we have clarify the sentence.

**Action by the authors:**

In our revised manuscript, we have carefully updated this part and modified it accordingly.

[Old Text] [Section “Introduction”, page 2, lines 31-33]

“In addition, about 30% and 20% of light can be trapped due to the surface plasmons means in organic layers and anode/organic waveguided modes in the anode layer [6].”

[Modified Text] [Section “Introduction”, page 2, lines 35-37 (Revised manuscript)]

“In addition, about 30% and 20% of light can be trapped due to the surface plasmons means in organic layers and anode/organic waveguided modes in the anode layer, respectively [5].”

**Reviewer #2 comment 4:**

*How stable is the OLED? Please comment it.*

**Response:**

We thank the reviewer for this comment. Several factors affect the performance of an OLED, and typically, there is never an ideal solution to obtain an optimized device in all relevant parameters. A compromise between stability and efficiency (i.e., EQE) is inevitable. However, with an appropriate material design, it is possible to achieve devices that balance these individual limitations. The goal of simulations is to predict physical properties from chemical composition, also known as the forward problem; this would allow for pre-screening and an overall better insight. Simulations can not only be advantageous but also are an essential tool in the design of future OLEDs [4]. The functionality of each layer is determined by electronic properties, optical properties, and depends on the choice of organic materials. The individual choice of each layer is important in enhancing the OLED in its entirety [4]. We selected the materials with refractive index and lattice constant matching for each of the layers and performed a lot of simulations to finalize the materials for different layers. In addition, α-NPD and Alq_3_ were considered as the donor i.e., electron-contributing and acceptor i.e., electron-accepting material in the emissive layer which achieved the optimized stability and efficiency [8, 9]. Due to the use of 5G for mobile communication and applications, increasing the efficiency has achieved the main focusing for maximizing the device performance which was solely on improving device stability previously. Therefore, we believe that our designed OLED will be stable and provides maximum performance in the field of OLEDs. Finally, we hope that our explanation will satisfy the reviewer for accepting our manuscript.

**Action by the authors:**

In our revised manuscript, we have added information about the stability of the OLEDs.

[Added Text] [Section “Materials and Methods”, page 6, lines 203-208 (Revised manuscript)]

“As stability and efficiency of OLED device is important, we achieved the optimized stability and efficiency by considering α-NPD and Alq_3_ as the donor i.e., electron-contributing and acceptor i.e., electron-accepting material in the emissive layer of OLEDs, correspondingly. Thus, we believe that our designed OLED will be stable and provides maximum performance which will lead to a great contribution in the field of OLEDs.”

**REVIEWER #3**

**Reviewer #3 comment 1:**

*The authors proposed a unique method to improve light intensity and efficiency of white organic light emitting diodes (OLEDs) by engraving micro lens arrays (MLAs) on the outer face of the substrate layer. Technically, the authors employ the numerical method with apparent authority, and the results are interesting for the readers. However, the necessary physical mechanism and references are absent in the manuscript to explain the results. Therefore, I suggest accept this manuscript after major revision.*

**Response:**

We would like to express our sincere gratitude to the reviewer for appreciating our work and the positive comments. In our revised manuscript, we have carefully investigated concerns that are raised by the reviewer and modified accordingly.

**Reviewer #3 comment 2:**

*Please clarify in more detail the FDTD simulation setting, e.g., grid size, light source, perfectly matched layer (PML), polarization, and the width, height, and depth of layers used in Fig.2.*

**Response:**

We would like to thank the reviewer for pointing out this important observation. In the manuscript, we missed some of the key factors of the FDTD simulation setting. As suggested by the reviewer, we added the information of the FDTD simulation setting in the revised manuscript. However, we mentioned different physical properties such as material name, layer name, length, thickness, refractive index and lattice constant of the materials used in the different layers of the OLEDs in Table 1 in the original manuscript.

[Old Table (Table 1)] [Section “Materials and Methods”, page 5]

**Table 1** Physical properties of several layers of OLEDs. [10].

| Material | Layer name | Length  (µm) | Thickness  (µm) | Refractive Index | Lattice  Constant  (Å) |
| --- | --- | --- | --- | --- | --- |
| Al | Cathode | 10 | 0.15 | 1.59 | 4.046 |
| Alq_3_ | Electron-transport molecular layer (ETML) | 10 | 0.18 | 1.75 | a=13.5190  b=15.8550 c=18.7110 |
| α-NPD | Hole-transport  molecular layer (HTML) | 10 | 0.6 | 1.9 | a = 7.9125  c =4.78382 |
| ITO | Anode | 10 | 0.2 | 1.8 | 10.1247 |
| SiO_2_ | Substrate | 10 | 0.9 | 1.45 | a = 4.914  c = 5.405 |

We also mentioned simulation environment i.e., temperature, time step, and mesh size in the original manuscript. In addition, we modified the information about simulation setting as suggested by the reviewer.

**Action by the authors:**

In our revised manuscript, we have added and modified information about FDTD simulation setting as suggested by the reviewer.

[Old Text] [Section “Materials and Methods”, page 7, lines 258-259]

“The simulations were run at room temperature (25ºC) using an 832-second time step. A value of 0.038 was observed as the mesh delta of X (*µ*m), Y (*µ*m) and Z (*µ*m).”

[Modified Text] [Section “Materials and Methods”, page 9, lines 282-288 (Revised manuscript)]

“The simulations were run at room temperature (25ºC) using an 832-second time step. A value of 0.038 was observed as the mesh delta of X (*µ*m), Y (*µ*m) and Z (*µ*m). We considered a grid of 100 *µ*m length and 10 *µ*m width, isotropic waveguide, vertical input plane source, Gaussian modulated continuous wave light pulse, Anisotropic perfectly matched layer (APML), and Y-polarization to observe DFT results for conducting the simulation in OptiFDTD simulator.**”**

**Reviewer #3 comment 3:**

*EL intensity should be defined in the text.*

**Response:**

We would like to thank the reviewer for pointing out this important issue. We have revised the manuscript according to your observation.

**Action by the authors:**

In our revised manuscript, we have added the definition of EL intensity.

[Added Text] [Section “Introduction”, page 1, lines 8-11 (Revised manuscript)]

“Electroluminescence is the outcome of [radiative recombination](https://en.wikipedia.org/wiki/Radiative_recombination) between an [optical phenomenon](https://en.wikipedia.org/wiki/Optical_phenomenon) and [electrical phenomenon](https://en.wikipedia.org/wiki/Electrical_phenomenon) where materials radiate lights in response to the passage of an [electric current](https://en.wikipedia.org/wiki/Electric_current) and the intensity of the radiated lights in this event is called electroluminescence intensity.”

**Reviewer #3 comment 4:**

*On line 333-334, the mechanism of “all models of spherical planoconvex MLA engraved OLEDs significantly increased the EL light intensity compared to basic OLED” should be elucidated in more detail and cite the relevant references.*

**Response:**

We would like to thank the reviewer for this important comment.

**Action by the authors:**

In our revised manuscript, we have modified and added the required information and cited references.

[Modified Text] [Section “Results and Discussion”, page 13, lines 361-363 (Revised manuscript)]

“Therefore, all models of spherical planoconvex MLA engraved OLEDs significantly increased the EL light intensity compared to basic OLED model which produced the EL intensity of 10000 arbitrary units [4, 9, 32].” [references in this response letter will be as, 7, 11, 12]

[Added Text] [Section “Results and Discussion”, page 13, lines 363-367 (Revised manuscript)]

“The above phenomena occurred because with the incorporation of spherical planoconvex MLA on the outer surface of OLEDs, curvature shape of the lenses confirms the smaller maximum incident light angles compared to critical angles and led to the light to be refracted rather than reflection.”

**Reviewer #3 comment 5:**

*The advantage of the proposed structure should be discussed in more detail and compared to other type of approaches, e.g., “Localized surface plasmon resonance enhanced by the light-scattering property of silver nanoparticles for improved luminescence of polymer light-emitting diodes” (see Journal of Industrial and Engineering Chemistry, 2021, 103, pp. 283–291).*

**Response:**

We would like to thank the reviewer for this comment. We discussed the advantage of our proposed approach in the original manuscript. In addition, we added the information of the above important paper as suggested by the reviewer.

**Action by the authors:**

In our revised manuscript, we have added the information and cited the above reference. In addition, we have modified and added information and advantage based on our proposed method.

[Added Text] [Section “Introduction”, page 3, lines 71-73 (Revised manuscript)]

C-L Huang et al. used photochemical reduction method for the modulation of the shape of silver nanoparticles and improved the efficiency of the polymer light-emitting diodes [24].

[Old Text + Modified Text] [Section “Introduction”, page 3, lines 93-106 (Revised manuscript)]

In this work, we incorporated an array of planoconvex and planoconcave lenses at the substrate layer and achieved high EQE and electroluminescence (EL) light intensity. We selected proper materials for each layer of the OLEDs considering refractive index and lattice constant matching among the adjacent layers. The produced OLED lights reflected backwards onto the OLED device from the interface of air/substrate because of TIR. This kind of behavior of OLED lights occurred since substrate has higher refractive index compared to air. Consequently, we engraved micro lens arrays on the substrate layer to reduce the TIR. The incorporation of MLAs on the outer surface accelerates the emitted lights from the surface to intrude with each other and create constructive patterns and destructive patterns in numerous locations. These patterns and distance between them reveal the smoothness and light spreading of the generated OLEDs. Therefore, various shapes and sizes of micro lenses have different impacts on the constructive and destructive patterns. In addition, for the amalgamation of MLAs on the OLEDs, maximum incident angles of light became smaller compared to the critical angle because of curvature shape of the lenses on the substrate layer and led the generated light to be refracted from the outer surface. As a result, EQE and intensity of OLED lights can be increased. We used FDTD (finite difference time domain) method for designing OLEDs. The simulation results confirmed that our proposed technique can produce a EQE of 17.30% for the Convex-1 elliptical planoconvex lens engraved OLED. In addition, EL light intensity is significantly increased for Convex-1 MLA engraved OLED compared to basic OLED. Outstanding light distribution and intensity in the visible range are observed for Convex-1 MLA engraved OLED which guarantees the production of white OLED. We believe that our proposed light intensity and efficiency improvement method will be applicable for the production of efficient OLED.

**Reviewer #3 comment 6:**

*Please briefly address the fabrication issue of the proposed structure and cite the related articles.*

**Response:**

We would like to thank the reviewer for the comment. We know that experimental results (fabrication) will give more clear idea about the performance of the designed OLEDs than the simulation results. Unfortunately, we don’t have any infrastructure to fabricate OLEDs in our laboratory. We will try to fabricate such kind of OLEDs in future, depending on the availability of appropriate equipment. However, goal of simulations is to predict physical properties from chemical composition, also known as the forward problem; this would allow for pre-screening and an overall better insight. Simulations can not only be advantageous but also are an essential tool in the design of future OLEDs [4]. We hope that the reviewer will understand our limitation and consider the simulation works for publication in the journal.

**Reviewer #3 comment 7:**

*The scale of color bar used in Figs. 5,6,7,9,10, 12,13,15, 16, should be the same and indicated the max. and min. values of color bars. One color bar used in one figure is enough.*

**Response:**

We would like to thank the reviewer for the comment. We obtained 3D light distribution, and 2D image map after completing the simulations for each of the models. We obtained the results from the FDTD simulations and found individual color bar for 3D light distribution, and 2D image map. Thus, we obtained two types of color bar for our simulations; one is for 3D light distribution, and another is for 2D image map. We represented the complete simulations results for each of the models including the color bar for all models. In our opinion, all the color bars of 3D light distribution are equal, and all the other color bars are equal for 2D image map. We hope that our explanation will satisfy the reviewer.

**Modified Text:**

**We modified a sentence to correct the actual meaning of the sentence.**

[Old Text] [Section “Results and Discussion”, page 22-23, lines 527-529]

“For the MLA etched OLEDs, most of the incident angles of light were smaller than the critical angle, making it possible to add curvature lenses to the substrate layer, which led to most of the generated light being refracted from the outside surface.”

[Modified Text] [Section “Results and Discussion”, page 22, lines 558-560 (Revised manuscript)]

“For the MLA etched OLEDs, most of the incident angles of light were smaller than the critical angle, because of curvature shape of the lenses, which led to most of the generated light being refracted from the outside surface.”

**REFERENCES**

# [1] [Dongmin Kim](https://www.pnas.org/doi/full/10.1073/pnas.2007395117#con1) et al., “Ultraflexible organic light-emitting diodes for optogenetic nerve stimulation,” PNAS, 117 (35) 21138-21146, 2020, <https://doi.org/10.1073/pnas.2007395117>.

[2] Yuwen Chen et al., “High efficiency blue and color-stable hybrid warm white organic light-emitting diodes based on a thermally activated delayed fluorescent material as an assistant host,” J. Mater. Chem. C, 8 (39) 13777-13785, 2020, <https://doi.org/10.1039/D0TC03644G>.

# [3] Bai Yu et al., “Higher Light Extraction Efficiency in Organic Light-Emitting Devices by Employing 2D Periodic Corrugation,” Frontiers in Chemistry, 9, 2022, 10.3389/fchem.2021.807867.

# [4] [Leanne Paterson](https://aip.scitation.org/author/Paterson%2C+Leanne) et al., “Computer aided design of stable and efficient OLED,” Journal of Applied Physics 128, 160901 (2020); <https://doi.org/10.1063/5.0022870>.

# [5] [CHANGYEONG JEONG](https://www.science.org/doi/10.1126/sciadv.abg0355#con1) et al., “Tackling light trapping in organic light-emitting diodes by complete elimination of waveguide modes,” *SCIENCE ADVANCES, 7 (26), 2021,* [DOI: 10.1126/sciadv.abg0355](https://doi.org/10.1126/sciadv.abg0355).

[6] Vidhi Mann and Vipul Rastogi, “Dielectric nanoparticles for the enhancement of OLED light extraction efficiency,” Optics Communications 387, pp. 202-207, 2017.

**[7]** A. Adhikary et al., “Improvement of light intensity and efficiency of n-ZnO/NiO/p-GaN heterojunction-based white light emitting diodes using micro/nano-lens array,” *Opt. Eng*. **59**, 117108 (2020) [[doi: 10.1117/1.OE.59.11.117108](a)].

[8] P. Heimel, A. Mondal, F. May, W. Kowalsky, C. Lennartz, D. Andrienko, and R. Lovrincic, “Unicolored phosphor-sensitized fluorescence for efficient and stable blue OLEDs,” Nat. Commun. **9**(1), 4990 (2018), <https://doi.org/10.1038/s41467-018-07432-2>.

[9] C. Eickhoff, P. Murer, T. Geßner, J. Birnstock, M. Kröger, Z. Choi, S. Watanabe, F. May, C. Lennartz, I. Stengel, I. Münster, K. Kahle, G. Wagenblast, and H. Mangold, “Long-lived and highly efficient green and blue phosphorescent emitters and device architectures for OLED displays,” in *Organic Light Emitting Materials and Devices XIX* (International Society for Optics and Photonics, 2015), Vol. 9566, p. 95662N, <https://doi.org/10.1117/12.2199079>.

[10] M. Polyanskiy, “Refractive index database,” https://refractiveindex.info (accessed 14 January 2022).

[11] M. S. Ahsan, M. R. Hossain, S. Mehbuba, K. M. Sarif Islam, M. Z. Amin and I. -B. Sohn, "Efficiency improvement of organic light emitting diodes (OLEDs) using micro/nano-structures," 2016 3rd International Conference on Electrical Engineering and Information Communication Technology (ICEEICT), 2016, pp. 1-6, doi: 10.1109/CEEICT.2016.7873069.

[12] B. Sana et. Al, “Micro/nano-structuring Assisted Efficiency Improvement of Organic Light Emitting Diodes,” 2021 8^th^ Brunei International Conference on Engineering and Technology (BICET), Universiti Teknologi Brunei, Brunei Darussalam, 2021
